# Using ways of knowing in nursing to develop educational strategies that support knowledge mobilization

**DOI:** 10.1002/pne2.12037

**Published:** 2020-09-07

**Authors:** Amelia Swift, Alison Twycross

**Affiliations:** ^1^ School of Nursing University of Birmingham Birmingham UK; ^2^ Open University Milton Keynes UK

**Keywords:** knowledge mobilization, nurse education, pain

## Abstract

There are continued challenges in achieving effective pain management for children and young people (CYP). Research has found several barriers to effective CYP pain management, which include, but are not limited to, deficiencies in knowledge among nurses and other healthcare professionals. Calls for improvements in and an increase in pain education ensue, in the expectation that an increase in knowledge will lead to an improved pain care for patients. Educational initiatives, as reported in the literature, have tended to focus on increasing empirical knowledge which has not resulted in the anticipated improvements in practice. An exploration of Carper's and Chinn & Kramer's five *ways of knowing* helps demonstrate why an over‐reliance on *empirics* fails to equip nurses for the realities of clinical practice and does not facilitate knowledge mobilization or improvements in pain care for CYP. In this paper, we explore these *ways of knowing* to produce a model for knowledge mobilization in (pain) education. Our model puts forward a multifaceted approach to education using the active learning principles which supports and equip nurses to become effective pain practitioners.

## 1 INTRODUCTION

Ensuring nurses have the knowledge to manage pain in children and young people (CYP) is important. However, there is increasing evidence that even when nurses have a good theoretical knowledge about how to treat and prevent pain in CYP this does not always result in improved practice.^1^ As there is evidence that pain in CYP is common,^2^, ^3^, ^4^, ^5^, ^6^ it is important to explore the reasons educational initiatives are not always successful. We believe this is due, at least in part, to the fact that the educational strategies used do not always facilitate the use of knowledge in practice. We also believe that nurses need greater support to apply their knowledge in practice.

Gary Rolfe developed what he called a new paradigm for nursing in the 1990s to address the *theory‐practice gap*, the difference between what we believe should happen based on theoretical and research evidence, and what actually happens. He argued that there was a misconception about the relationship between theory and practice.^7^ Theory is generated to explain what is observed, and then, it is used to predict what will happen in similar circumstances. Theory must always develop to improve that power of prediction, the development being stimulated by noticing that the theoretical model does not capture observed reality as well as it might. However, this *technical rationality* model has tended to separate the theorist from the practitioner, something recognized and addressed by Schön^8^ by relating Dewey’s *Theory of Inquiry* ^9^ to his own model of reflection.^10^ Schon speaks about the limitation of traditional knowledge as a means of solving complex real‐life problems and about how the development of a dichotomous approach to knowledge generation and application has inevitably led to a situation where that knowledge does not support effective in‐practice problem‐solving and growth. One of the central issues, he argues, is that of professionalism:

The systematic knowledge base of a profession is thought to have four essential properties. It is specialized, firmly bounded, scientific, and standardized.^10^

**Figure 1:**
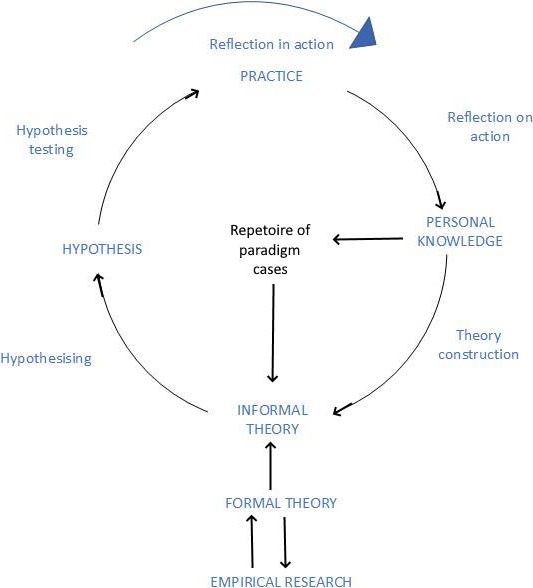
Rolfe's model for nursing praxis

The use of Schon's reflection‐in‐action and reflection‐on‐action allows the nurse to move beyond this notion of professional knowledge as a tool to be used by the *nurse technician*. They become *nurse practitioners* when they modify and generate knowledge based on their experiences and encounters in practice. Rolfe^7^ created a diagrammatic representation of his model for nursing praxis (Figure 1) which shows how formal knowledge feeds into an active process of experiential learning. While this model helps to explain how to close the gap generated by technical rationality and early notions of professionalism, it does not explore the role of the educator.

Nurse education has become overly content‐driven, focusing on teaching facts, processes, and skills^11^, what Sfard^12^ used the *acquisition metaphor* to describe. This approach to teaching and learning regards the student as a vessel that can be filled with facts and skills. We see this in overall curriculum design and delivery, which in many countries including the United Kingdom has to meet standards set by a regulatory body. In the UK, the Nursing and Midwifery Council (NMC) sets the educational standards. The document detailing the development of the most recent standards encourages an acquisitional approach. The standards describe “*what people will need nurses and midwives to know and be capable of doing…”*.^13^

The acquisitional approach is also encouraged by pain‐specific curricula and guidelines of which there are many (eg,. British Pain Society,^14^ European Pain Federation,^15^ International Association for the Study of Pain Educational Initiatives Sub‐group,^16^ Royal College of Nursing^17^) These documents detail essential, desirable, and specialized knowledge required for generalist and specialist practitioners from different health professions. Comparison of the documents shows a consistent baseline of empirical knowledge is recommended including pain physiology, assessment, different types of pain, management strategies, and special populations including CYP.

Demonstration that your program of study will deliver *what* nurses need to know leads us down a rabbit hole of listing content, which can then lead simply to the delivery of that content. This completely ignores the way knowledge is used in practice, creating a situation where knowledge can be good but patient outcomes poor. There is ample evidence of this being the case in children's pain management. Pain knowledge has been identified as good (with room for improvement) in some studies^18^, ^19^, ^20^ and poor in others.^21^, ^22^, ^23^, ^24^ Many studies demonstrate that various educational interventions can improve pain knowledge (see reviews Alotaibi et al.^25^, AlReshidi et al.^26^) but very few demonstrate that increased knowledge is sustained or that it improves patient outcomes. Indeed, the detachment of theory and practice is so ingrained that rarely are patient outcomes included in the evaluation of the success of the educational intervention. Only four studies within the last five years explored the effect of pain education on pediatric nurses’ pain management behaviors. Three studies showed improvements in analgesic drug prescribing^27^ ^,^ analgesic drug administration^28^ ^,^ and compliance with pain‐related palliative care clinical guidelines.^29^ The other study did not find any improvements in pain assessment behavior following an educational intervention.^19^

To improve pain management through education, we must change the way we view knowledge and focus on the end goal—confident, competent practitioners who can make safe and effective clinical decisions in the real world that lead to good patient outcomes. Moore et al^30^ proposed a model Box 1 that articulates potential outcomes of continuing education against which any educational strategy can be measured. The goal of education in terms of knowledge mobilization is, therefore, to support students in achieving Level 5 and beyond.

BOX 1 **Moore et al^30^ framework for continuing education outcomes**

Level 1: Participation or attendance.

Level 2: Learner satisfaction.

Level 3: How far have learner expectations been met.

Level 4: Demonstration of competence in the knowledge transmitted within the educational environment.

Level 5: Ability to translate the knowledge or skill into performance in practice.

Level 6: Demonstration of benefit to the patient as a result of the student's actions.

Level 7: Demonstration of change in the health status of the community.

Knowledge mobilization is not about addressing a theory‐practice gap. Using that term perpetuates the notion that there is something missing that prevents the application of empirical knowledge in practice. Once *knowledge* is accepted as being multifaceted, we can see how the expectation of application is overconfident. Education needs to be multifaceted to equip students to practice in the real and complex world. In this paper, we will draw on Carper's^31^ *ways of knowing* in nursing together with Chinn and Kramer's concept of *emancipatory knowing* (2010). We will use these ways of knowing to develop a different understanding of what education is needed in order to mobilize factual knowledge into effective practice.

## 2 WAYS OF KNOWING AND ITS APPLICATION TO PAIN IN CYP

Carper's^31^ seminal work outlines *ways of knowing* in nursing. The four ways of knowing are *empirics—the science of nursing, esthetics—the art of nursing, the component of personal knowledge in nursing,* and *ethics—the component of moral knowledge in nursing*. Chinn and Kramer^32^ added to Carper's model proposing a fifth and overarching type of knowing, *emancipatory knowing*. This is about understanding societal issues and being able to take action to create both personal benefit for the patient using this knowledge and change at a societal level.

### 2.1 Empirical knowing

*Empirical knowing* is the science of nursing, which is factual, descriptive, and helps to develop abstract and theoretical explanations. Nurses demonstrate empiric knowing on a practice level through the competent performance of activities supported by theory.^33^ Empirics is concerned with questions such as “what is this?” and “how does this work?”. This kind of knowledge is often organized using models and theories allowing generalization from the specific.

An example of the importance of empirical knowing in pediatric pain management is the biopsychosocial model. Chronic pain in CYP is multifactorial with a complex interplay between biology, psychology, and sociology. Vetter et al^34^ developed a multidimensional model of pediatric pain to describe the interplay between myriad factors with the aim of enhancing the practitioners understanding of the effects of chronic pain on the child and parents. The model is grounded in scientific study—justifiable and defensible facts. Understanding this model, and appreciating the investigation behind it, is intended to help nurses to understand what they observe and to act upon it.

In terms of pain management, the empirical way of knowing has taken priority in terms of the education offered to pre‐ and post‐registration nurses. However, studies demonstrate that even with good technical knowledge there would still be barriers to effective pain management. Twycross^35^ reported that children sometimes refused to take analgesic drugs offered to them. Other barriers relate to a lack of cooperation by parents^36^ ^,^ although this is not a universally supported finding.^37^ A frequent finding in pediatric pain research is the need for improved communication and emotional support for the parents.^38^, ^39^ These findings show how nurses’ ability to build a good working relationship with CYP and parents is vital, and how within that relationship the nurse must be a listener, nurturer, teacher, and information source. While knowledge of pain physiology or medication is a requirement to fulfill these roles, it is only a part of what is required.

Other barriers to effective pain management include poor prescribing practices and limited access to medications,^40^ a wish to hand over care and decisions to pain specialists^41^, and a lack of such specialists.^42^ There are also challenges in terms of healthcare funding, service configuration, workload pressures,^43^ as well as interprofessional communication and teamwork.^44^ It is clearly not enough to have the empirical knowledge about what to do to achieve effective pain management. It is also important to consider the context in which care is taking place, and the social, psychological, ethical, and professional consequences of actions and omissions.

Schon (1992) felt a reliance on empirical knowing failed to equip students to work with real‐world uncertainty. He felt that the most competent practitioners were not necessarily the most “knowledgeable” but those who had talent, wisdom, intuition, or artistry. Empirical knowledge never stands alone because in practice nurses use various other *ways of knowing* to determine the right course of action.

### 2.2 Esthetic knowing

*Esthetic* knowing is often described as the *art of nursing*. Chinn and Kramer^32^ describe this as a way a nurse can form a sense of patients’ needs and act on their behalf, taking into account nuanced information about their personality and previous experiences to orchestrate the most effective way of providing care.

The art of nursing draws on other ways of knowing: personal knowledge; experience; reflection; ethics; and empirical information and aims to help the nurse to develop a practical wisdom. Bowdoin^45^ describes this as thinking like a nurse, which she explains comprises four attributes: critical thinking, clinical judgment, moral reasoning, and professional competence. These attributes allow nurses to finesse their actions (nursing care) to produce the most acceptable treatment path for the patient with the aim of achieving the optimum outcome. In the context of pediatric pain management, this is about providing care that considers the views of CYP and their parents as well as engaging in shared decision‐making. Nurses schooled in this way of knowing consider nursing care as something that encapsulates the CYP and parents’ resources in the widest context, while also considering issues of sustainability and public health.

### 2.3 Ethical knowing

*Ethical knowing* enables the practitioner to consider the morals associated with choices in treatment for themselves and the patient. This goes beyond what professional codes of conduct (eg, the UK Nursing and Midwifery Council^46^) state ought to be done, or what one is obliged to do. Ethical knowing also includes the ability to evaluate motives, values, character, and norms.

As we move from childhood to adulthood, our moral decision‐making changes.^47^ We begin with an extrinsically driven desire to avoid punishment or to receive a reward. Next, we explore how to follow societal rules and fit in. Later, we are able to explore moral decision‐making in complex contexts, making decisions that may go against moral norms. Nurses’ self‐construct, no matter our culture or gender, includes a desire and capacity to care.^48^ Thus, we are not entirely driven in our moral reasoning by justice (Kohlberg's view) but also by the ethics of care.^49^, ^50^

Pain is an example of a condition that complicates moral reasoning. Nurses are ethically driven to connect with the distress and fear that a CYP in pain experiences. Seeing a CYP suffer evokes an emotional response in nurses that can influence decisions made,^51^, ^52^ but decisions must be made within an organizational context. This can preference management decisions, economics, and technology above human relationships and moral decisions.^53^ Often, nurses need to prioritize other tasks above pain management,^54^, ^55^ and missed care is associated with moral distress.^5657^

Nurses must also navigate the ethical tensions that arise when what can be achieved may be at odds with they feel should be achieved, for example, the tension between increasing a CYP’s suffering by giving them a new, untested treatment, and the potential, however slight, for a cure,^58^ or caring for a child conceived to be stem cell or bone marrow donors, who will experience pain and other consequences of the surgery,^59^, ^60^ or the US federal regulation that states that CYP cannot be provided with hospice care at the end of life if they are receiving any form of “curative” treatment.^61^

### 2.4 Personal knowing

The *personal way of knowing* is the knowledge we have of ourselves and knowledge of how this colors our interactions with others and encompasses experiential learning. Dewey^62^ noted that experience is not in itself educative. To be educational, an experience must be analyzed or reflected upon. Schon^63^ was an early proponent of reflection, particularly reflection‐in‐action—*thinking about what they are doing while they are doing it*.

Personal experience of pain appears to help nurses have better empirical knowledge^64^ and motivation to manage pain.^65^ Motivation to manage pain is central to nurses’ role as a carer and is based on the value of compassion. Compassion is defined as:

The good which moves or motivates a nurse to act in a manner which helps in alleviating the suffering of another.^66^

Hence, compassion has a moral quality to it, and its manifestation can be made more difficult because of myriad demands on nurses’ time, and the influence of others whose priorities the nurse might not share. Jones et al^66^ describe this as a *con‐fusion of horizons*. The horizon is akin to our standpoint, it is individual and formed of our experiences, education, culture, language, and more. The horizon describes our ability to see beyond what is immediately in front of us. Ideally, the practice of nursing would take place in a space in which the horizons of all staff are the same (fused)—a fusion of horizons. However, horizons do not fuse and this *con‐fusion* leads to moral distress and compassion fatigue, which Jones et al^66^ suggest can be remedied by opening up a dialogue, speaking out, and working through daily conflicts and challenges. Thus, nurses need skills to be able to perceive their feelings, articulate them, create a safe space, and work through issues.

### 2.5 Emancipatory knowing

*Emancipatory knowing* prepares nurses to advocate for social justice and human rights on behalf of their patients and in a wider context. This way of knowing originated from the development of a *Nursing Manifesto* ^67^ which critiqued traditional nursing practice and its tendency toward the medical model. The authors argued that nursing and health care should be reformed to enhance health and quality of life for everyone.^68^ Nurses should be at the forefront of driving such change.^69^

The ideas, values, and worldviews behind the *Manifesto* describe the scope of emancipatory knowing and of the activism nurses should, ideally, engage in. This encourages nurses to view their practice in a global context, to value diversity, to be inclusive, optimistic, and community minded. Nurses should also practice ethically, holistically, and critically—challenging and changing traditional practice to create a more socially just and participatory healthcare system. The *Manifesto* calls on nurses to become activists. However, to date nurses have not committed to this in great numbers^70^ and educators are ambivalent or unsure how to go about supporting students’ development in this context.^71^

There are many examples related to pediatric pain management affected by injustice, inequality, political and policy decisions and human rights. Nurses’ voice in this context could, and many argue should, be influential. For example, forced migration and asylum seeking is increasing with many CYP experiencing health problems including pain.^72^, ^73^, ^74^ Pain is related to health inequality. For example, orofacial pain is more prevalent in socially deprived areas of even high‐income countries.^75^, ^76^ Student nurses tend to have positive attitudes toward diversity, inclusivity, and social justice^77^ ^,^ but there are relatively few that are politically active^71^, ^78^ and limited exploration of this aspect of content‐driven curricula that are designed around technical knowledge acquisition.

In the context of CYP pain management, the nurse provides support and care to the family, not just the patient. The family will have additional concerns and needs above and beyond those affecting the patient. For example, a nurse may be supporting a family to provide complex care for one CYP, while they are traveling on public transport to frequent hospital appointments and caring for school‐aged siblings at different schools. Many will be doing so with the added burden of a precarious financial and housing situation. Reflection on this can help nurses become more aware of the wider community context, enabling them to support the development of effective services for the many. Increasingly, students are not just required to employ emancipatory knowing in relation to individual patients and their families but need to consider the way health service developments impact on our ability to provide care at a societal level. For example, disparities in health problems and access to services vary significantly across different parts of a city and between urban and rural environments.

In this section, we have explored the ways of knowing that are fundamental to nurses being able to make competent and confident decisions about pediatric pain management. We have shown how the knowledge required for this role goes beyond empirics and encompasses the art and science of nursing in its broadest sense. In the next section, we translate these ways of knowing into a model for teaching nursing and use the example of pain management for CYP nursing as an example.

## 3 THE WAYS OF KNOWING PAIN MODEL

**Figure 2:**
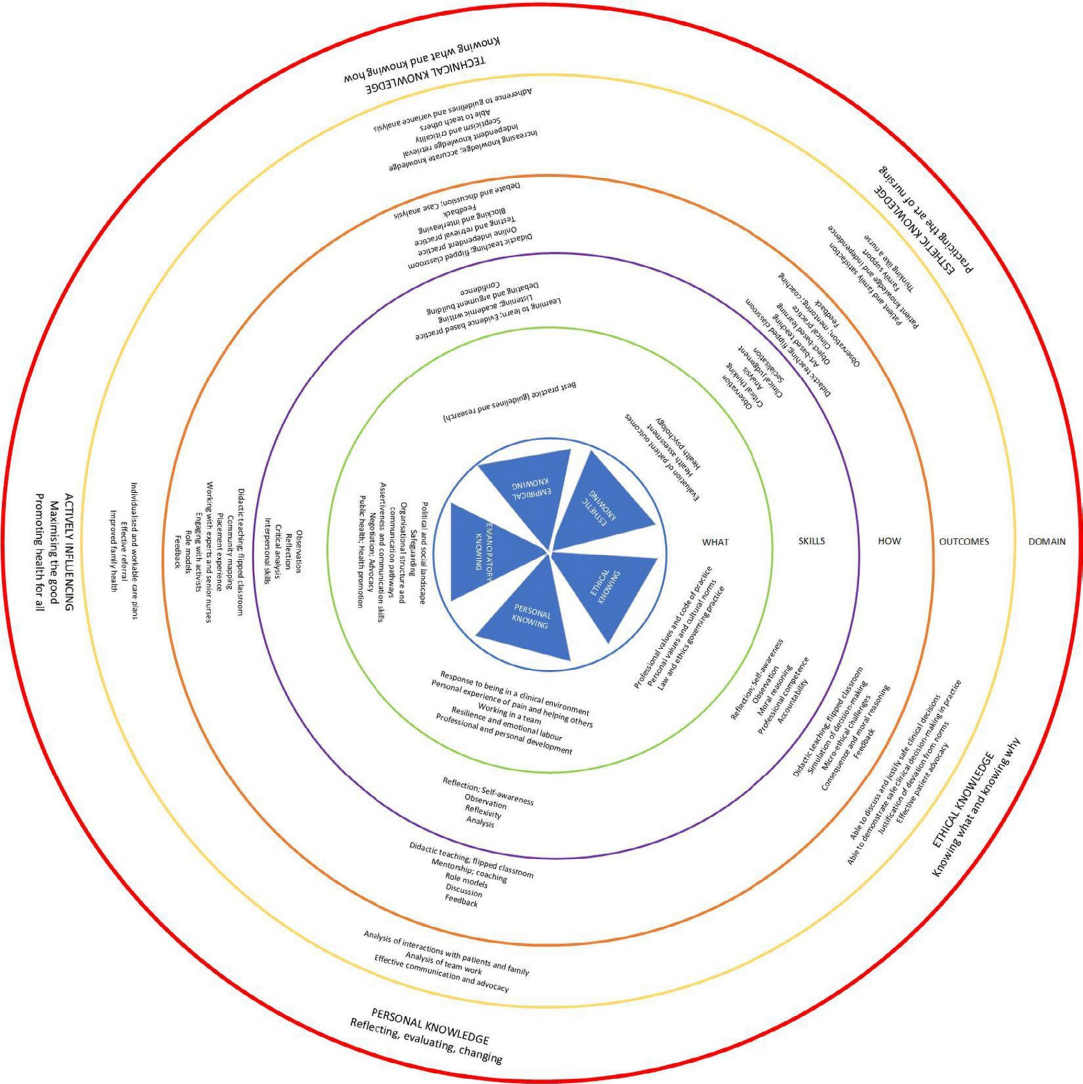
Ways of Knowing Pain

Each of the ways of knowing described above offers a component of what the nurse needs to become an autonomous pain practitioner, which is to say that they could use knowledge to observe, interpret, and solve CYP pain problems. A range of teaching and learning strategies would be required to support this development. Our *Ways of Knowing Pain* model is presented in Figure 2.

The innermost area provides the *Ways of Knowing* to which the rest of the model relates. Our contention is that failing to address all of the *ways of knowing* means that student education is incomplete. Students will only have some of the knowledge and skills required to mobilize knowledge, that is, to make use of knowledge in the effective care of patients.

The next layer of the model further articulates the *way of knowing*, relating it to the essential knowledge a nurse must have, we have called this the *what* of knowledge, as in “what does the nurse need to know.” The third concentric circle refers to the skills the nurse needs to have to be able to act on the “what” and to take advantage of the “how.” The *how* refers to teaching and learning strategies with proven effectiveness. For example, in the esthetic way of knowing the student is learning to evaluate patient outcomes, which requires skills in observation, which can be taught by a range of methods including arts and object‐based learning.^79^ The next layer, the penultimate, refers to the expected outcomes of the learning activity—that is, what we expect to see in terms of student attainment and behaviors. Articulating clearly what you expect the student to be able to do (demonstrate) in practice completes the exercise of knowledge mobilization. If we do not consider nurse behaviors and their links to patient outcomes, we are unable to evaluate the teaching and learning in practice and are back to *square one* with our efforts to teach not resulting in competent or proficient practitioners. The outermost circle articulates the domain of learning in the shared language of nurse education.

### 3.1 Application of the ways of knowing pain model to a case study

A student nurse embarks on a journey of learning that begins before they enter a formal program of study. Thus, each student comes with experiences, knowledge, values, beliefs, and expectations. The role of nurse education is to maximize the knowledge, skills, and attributes that the student already has and to help them acquire and develop more so that they become capable of independently providing safe and effective care in a variety of environments. Often this transition is described using Benner's model of novice to expert.^80^ In this case study, we will explore the case of a novice student who has no clinical experience being prepared to undertake a postoperative pain assessment on a young child whose parents are present. While some of the teaching that takes place is specific to pain management, other aspects apply to all nursing practice. However, we contend that to achieve the best results the student would have the opportunity to use pain‐specific examples in simulation and theory sessions before being supported and coached to use these strategies in clinical practice.

Table 1 divides a number of educational activities and outcomes into the five ways of knowing with suggestions of what content, skills, and outcomes could be used to support the students’ development of a rounded knowledge set that can be applied in practice. Further, there are suggested outcomes that could be used to evaluate the development of knowledge. The utility of this model becomes more apparent when we try to construct learning activities using it. The empirical content is relatively easy to determine but the real benefit to the student is considering how that can be made use of in the practice setting. When consideration is given to the other ways of knowing, we can begin to appreciate that knowing what pain assessment is cannot translate into performing that activity well in a complex real‐life situation because there are a host of other skills required.

**Table 1:** Case study

	What	Skills	How	Outcomes
Empirical knowing	Mechanism of postoperative pain. Pharmacological management of pain. Assessment of acute pain.	Knowledge acquisition. Formulating and asking questions. Use of bibliographic databases. Documentation and record keeping.	Lecture. Quiz. Video case study with guided note taking. Simulation Role modeling and coaching in clinical practice. Supervised practice and collection of patient outcome evidence.	Knowledge growth (quiz, written assignment, poster development). Demonstration of skills in practice.
Esthetic knowing	Factors that influence the child's pain and response to management strategies. Parental involvement in pain management and response to their child in pain	Observation of practice and recording observations. Having sensitive conversations. How to talk to parent and children.	Use of evidence based checklist of known barriers to effective pain management (students can prepare this if there is time available). Reflection on practice.	Well‐constructed written or audio reflection meeting‐specified criteria.
Ethical knowing	Legal and ethical responsibility of the nurse in relation to pain management.	Observation. Debating and discussion. Problem‐solving.	Observations skills using fine arts. In class or online debate using a case study.	Participation in synchronous or asynchronous discussion or debate.
Personal knowing	Emotional labor associated with the suffering of others.	Reflection as an informal and formal skill. Academic writing. Projecting confidence and developing confidence. Communication with powerful.	Simulation. Reflection.	Well‐constructed written or audio reflection meeting‐specified criteria.
Emancipatory knowing	How does the organizations goals influence the work of the nurse in addressing the pain management needs of the individual patient? Targets and metrics.	Systematic searching. Navigation and understanding of the local healthcare organization available metrics. Confident communication. Preparation to interview senior staff members. Time management. Picking the right time. Coping with rejection and coming back for more.	Exploration by finding and discussion of local policies, CQC reports, dashboard metrics. Discussion with senior nursing staff.	Well‐constructed written or audio reflection meeting‐specified criteria. Portfolio entries.

## 4 CONCLUSION

Pain management for CYP is suboptimal. This is often blamed on *poor knowledge*, and it is proposed that this can be addressed by improving pain education. We have demonstrated that poor knowledge is more complex than much research suggests. Knowledge is not just empirics, and treating it as such means that the solution of providing yet more empirical knowledge, or filling the empty vessel, cannot address the real issues. Knowledge is multifactorial, and education should take this into account.
